# Internal Medicine

**Published:** 1913-01

**Authors:** 


### Books and Magazines.

INTERNAL MEDICINE. By David Bovaird, Jr., A. B., M. D., assistant professor of clinical medicine in the College of Physicians and Surgeons of Columbia University, etc., etc. Octavo, 618 pages, illustrated. J. B. Lippincott & Co., Philadelphia and London, 1912. Price, $5.

This is an exceedingly interesting book of 618 pages, written by an able and an experienced teacher in one of the oldest, most thorough and up-to-date medical schools in America; and it embodies, in an easy reading and highly condensed form, what the author regards as "the more important facts of the subject of internal medicine," which the specialists have left of the older "practice of medicine."

The enviable reputation which Dr. Bovaird enjoys as a teacher will be enhanced by that which the present volume will add to it as an author, for he has done his work well and has given to the practician, the student and the nurse, in 581 octavo pages, about all that is *really known* of some 500 or more subjects which it discusses. It cannot be charged that the author has given undue prominence to any one subject. The converse, however, cannot be truthfully said. Pellagra, for example, a disease that has caused over 60,000 deaths in the United States within the last few years, and is daily increasing, cannot be properly treated of in the space —a little over one page—dedicated to it in this work, nor can uncinariasis, a disease epidemic in many parts of the country, in the two pages allotted to its discussion. Such defects, however, are not fatal, for the work is an excellent one and will meet the need for which it was written as fully as could be expected in one of its pretensions, and should be in the library of every practicing physician and in the hands of every student and nurse desiring a *"multum in parvo"* that is up to date. The appendix alone is worth the price of the book.

The 109 engravings and seven colored plates which illustrate the volume are excellent and timely, as are also the paper, type and binding, qualities which characterize the products from Lippincott's house. R. H. L. B.

